# Middle-aged and older people’s preference for medical-elderly care integrated institutions in China: a discrete choice experiment study

**DOI:** 10.1186/s12912-023-01696-w

**Published:** 2024-01-10

**Authors:** Mao-min Jiang, Mei-fang Xiao, Jia-wen Zhang, Mei-fang Yang

**Affiliations:** 1 School of Public Affairs, Xiamen University, Xiamen, Fujian province China; 2 School of Nursing, Gannan Medical University, Ganzhou, Jiangxi province China; 3 Xiamen Institute of Software Technology, Xiamen, China Fujian province; 4 School of Education, Silliman University, Negros Oriental province, Dumaguete, Philippines; 5 School of Nursing, Southwest Medical University, Luzhou, Sichuan province China

**Keywords:** Aging, Middle-aged and Elderly People, Medical-Elderly Care Integrated Institutions, Discrete Choice Experiment, Preferences, Quality of Life

## Abstract

**Background:**

With the continuing impact of the aging population, medical-elderly care integrated institutions, as a way to bear the pressure of medical and elderly care, effectively ensure the quality of life of the elderly in their later years.

**Objectives:**

To explore the preferences of medical-elderly care integrated institutions among Chinese middle-aged and older people and to provide a reference for establishing elderly-oriented development of medical-elderly care integrated institutions.

**Methods:**

In this study, a discrete choice experiment (DCE) was used to investigate the preferences of people aged 45 years and older in medical-elderly care integrated institutions in China from October 20, 2022, to November 10, 2022. A mixed logit regression model was used to analyze the DCE data. Participants’ willingness to pay for each attribute was also calculated.

**Results:**

Data from 420 participants who provided valid responses were included in the analysis. In terms of the choice preference, moderate service quality (vs. poor service quality: *β* = 1.707, *p* < 0.001, 95% *CI* 1.343 ~ 2.071) and high medical technology level (vs. low medical technology level: *β* = 1.535, *p* < 0.001, 95% *CI* 1.240 ~ 1.830) were the most important attributes to middle-aged and older people, followed by monthly cost, environmental facilities, the convenience of transportation, and entertainment activities. Regarding the willingness to pay, participants were more willing to pay for service quality and medical technology level than for other attributes. They were willing to pay $3156 and $2838 more for “poor service quality” and “low medical technology level,” respectively, to receive “moderate service quality " (*p* = 0.007, 95% *CI* 963 ~ 5349) and “high medical technology level” (*p* = 0.005, 95% *CI* 852 ~ 4824).

**Conclusions:**

The state should attach great importance to the development of medical-elderly care integrated services industry, actively optimize the model of the medical-elderly care integrated service, improve the facilities, and create a healthy environment. At the same time, give full play to the role of medical insurance, long-term care insurance, and commercial insurance, so as to improve the comprehensive quality of life of the elderly.

**Public contribution:**

The design of the experimental selection was guided by 10 experts in the field, 5 Chinese government officials, and interviews and focus group discussions, without whose participation this study would not have been possible.

**Supplementary Information:**

The online version contains supplementary material available at 10.1186/s12912-023-01696-w.

## Introduction

The aging of the population has become a significant trend in the 21st-century human social development, with the global number and proportion of elderly individuals increasing. According to publicly available data from the World Health Organization (2022), the global population aged 60 and above is estimated to reach 1.4 billion by 2030 and is projected to increase to 2.1 billion by 2050 [1]. In China, individuals aged 65 and above constitute approximately 13.50% of the total population, with over 40 million disabled elderly individuals, accounting for around 21.05% of the elderly population [2]. With the improvement of medical technology, the average life expectancy is increasing year by year, and the number of older people with care requirements is increasing, which greatly impacts the national elderly care system and health system [3–4]. In order to cope with the demand for elderly care services brought by the aging population, the Chinese government attaches great importance to the development of the elderly care service industry, actively promotes the medical-elderly care integrated service model, optimizes the elderly care structural system, and continuously creates high-quality elderly care service products [5–6]. As a comprehensive elderly care service model to address healthy aging and improve the quality of life of the elderly, the integration of medical and elderly care services has become a critical systematic arrangement to promote the health China strategy [7–8].

In order to fulfill the diverse needs of older people, the Chinese government has carried out systematic elderly care services based on home care, relying on community care, and supplemented by institutional care [9–10]. With the increasing number of older people with disabilities and dementia, the demand for medical-elderly care integrated institutions with professional medical care services is also increasing [10–13]. Medical-elderly care integrated institutions are medical and health institutions or nursing care institutions that have both medical and health care qualifications and nursing care service capabilities to provide integrated nursing care services for the elderly, including medical care, nursing care, rehabilitation, health management, and hospice care [14–15]. A professional medical team can enable the elderly to receive timely and professional healthcare in nursing homes, reducing the probability of them going out for medical treatment and greatly alleviating strained medical resources [16]. Systematic nursing services can provide a comprehensive understanding of the health status of the elderly and provide psychological and health guidance for the elderly in a well-rounded and personalized manner [17]. Medical-elderly care integrated institutions can not only improve the quality of life of the elderly but also alleviate family conflicts [18].

Currently, the development of medical-elderly care integrated institutions is immature, and there are still many problems. Some studies have found that the slow development of medical-elderly care integrated institutions is due to the lack of supportive policy of medical care and senior service integration and the problems caused by the cross-management of multi-departments [19]. Secondly, most elderly care institutions only provide general life care or are only equipped with simple medical facilities, which cannot meet the medical care requirements of the elderly [20–21]. In terms of service quality, there is a lack of professional nursing personnel and a severe imbalance in the ratio of nursing personnel to the elderly [22–23]. Related studies also found that the quality of nursing personnel in some elderly care institutions is generally low, and the service attitudes need to be improved [24–25]. Most of the existing medical-elderly care integrated institutions are mainly elderly care institutions with embedded medical institutions, and the career development of doctors is highly restricted [26–27]. Therefore, there are difficulties in forming professional medical teams, and the technical level of doctors could be higher [28]. At the same time, the monthly costs are also relatively high due to the ample space occupied by elderly care institutions, most of which are located in remote areas, which in turn dramatically restricts the development of medical-elderly care integrated institutions [29–31].

The development of medical-elderly care integrated institutions cannot be separated from the preferences of the elderly and their families. A study found that specialized medical and precise care services are essential factors in improving the satisfaction of elderly care institutions [32–33]. Another study showed that compared with low-end and high-end, mid-end elderly care institutions with moderate prices and complete facilities and services are more preferred by the elderly and their families, and the lower service costs and personalized service approach can meet the needs of most older people’s care services [34]. A related study found that older people of higher age have higher requirements for medical and health facilities and the environment of elderly care institutions. In contrast, the lower-aged elderly prefers spiritual comfort and rich cultural and entertainment activities [35–37]. There are significant differences in the preferences of older people and their family members in choosing elderly care institutions, specifically in the level of medical care, environmental facilities, spiritual culture, and service costs.

In summary, both domestic and international perspectives on the concept of integrating medical and elderly care aim to harmonize medical and elderly care services for a more comprehensive fulfillment of the health needs of the elderly. These discussions have delved into strategies for delivering medical services within elderly care institutions and seamlessly integrating medical and non-medical services into a comprehensive healthcare model. Emphasis has been placed on products and services within the elderly care sector, particularly the application of smart technologies in institutions dedicated to the integration of medical and elderly care. This encompasses aspects like remote medical monitoring, smart health devices, and AI-assisted diagnostics. Furthermore, research has been conducted on the policy and regulatory framework governing institutions that integrate medical and elderly care. This research addresses critical issues such as government support, fund allocation, and regulatory mechanisms. Currently, there is limited global research on the selection preferences for medical-elderly care integrated institutions. Existing studies primarily focus on aligning the supply of elderly care institutions with the needs of the elderly. Only a comprehensive understanding of the preferences of the elderly and their families for medical-elderly care integrated institutions can inform targeted improvements in service content and methods. Hence, this study concentrates on discerning the choice preferences of Chinese middle-aged and older individuals regarding medical-elderly care integrated institutions through discrete choice experiments (DCE). The objective is to provide insights for the establishment of medical-elderly care integrated institutions that cater to the specific needs of the aging population.

## Methods

A discrete choice experiment (DCE) was used to explore the choice preferences of middle-aged and older people for medical-elderly care integrated institutions. Two alternatives were provided in each choice set of this experiment, and participants could choose one of them according to their thoughts. To ensure that each participant made a choice and learned about their preferences for institutional attribute settings, we did not provide an exit option, and Table 1 shows the example choice set.

**Table 1:** An example of choice set

Attributes	Choice Set A	Choice Set B
Environmental Facilities	Moderate	Good
Service Quality	Moderate	Good
Medical Technology Level	Moderate	Low
Entertainment Activities	General	Not rich
Convenience of Transportation	10 ~ 30 min	10 ~ 30 min
Monthly Cost	$297	$559
**Which one is more attractive**	□	□

### Selection of attributes and levels

According to the definition of DCE, scientificity and rationality are required in setting the attributes and levels. Participants also need to be familiar with the content of choice set for different combinations. Also, the design should take complete account of the inhibitory nature of the levels to ensure that participants can weigh the most preferred choice set for different combinations [38]. In the domain of health, the number of attributes is typically 4–6 [39], and the ideal number of choice sets is 8–16 [40].

First, the research team mainly combed the literature from Web of Science, PubMed, China national knowledge infrastructure (CNKI), and other databases and conducted a systematic review, and then used government work reports and relevant government regulations across China to explore the attitudes and factors influencing the choice preferences for elderly care institutions among middle-aged and older people. Secondly, based on the literature analysis, we conducted 5 one-on-one and 3 focus group interviews. Then we invited 10 experts in related fields (public administration major, public health major, psychology major, sociology major) and 5 government officials (Civil Affairs Bureau, Health Bureau, Committee on Aging) for an expert consultation. Finally, we determined 6 attributes, each containing 3–4 levels. The details are shown in Table 2 and interview outline.

**Table 2:** Attributes and levels of discrete selection of medical-elderly integrated institutions

Attribute and level	Description	Paraphrase
Environmental Facilities		This includes the housing structure, physical facilities, and health environment of the elderly care institutions.
1	Poor	
2	Moderate	
3	Good	
Service Quality		Including the establishment of health records of the elderly, the service quality, service attitude and spiritual comfort of nursing staff, etc.
1	Poor	
2	Moderate	
3	Good	
Medical Technology Level		The number, title, treatment effect and emergency response capability of doctors in medical-elderly care integrated institutions.
1	Low	
2	Moderate	
3	High	
Entertainment Activities		Including the frequency of daily activities of the elderly, the diversity of activities, etc.
1	Nor rich	
2	General	
3	Very rich	
Convenience of Transportation		Walking time to the nearest bus stop
1	> 30 min	
2	10 ~ 30 min	
3	< 10 min	
Monthly Cost^*a*^		Average monthly cost of living in an medical-elderly care integrated institution
1	$297	
2	$559	
3	$838	
4	$1117	

^*a*^ Based on a currency exchange rate of 7.1598 yuan to $1.00 in November 2022

### DCE instrument design

The research team used a fractional factorial design method to determine the optimal number of choices for the medical-elderly care integrated institutions. If a complete factorial design is adopted, 972 combinations (35 × 4) will be generated under different attributes based on the study’s six attributes and 19 levels. The fractional factorial method is commonly used in the design of DCE choice sets based on two principles, the principle of orthogonality and the principle of balance [41–42]. Therefore, we completed the generation of choice set by orthogonal experiments in SPSS AU software, selecting L18.3.6.6.1 according to the orthogonal table manual and deleting scenario 1 according to the internal validity test, resulting in 17 scenarios and using scenario 9 as a control to form 16 choice sets. To avoid burdening the participants, we randomly split the 16 choice sets into 2 sets of 8 choice sets each by the random number method, and to assess the validity of the DCE questionnaire, we included a choice set with a clear advantage (lie detector questions) in both questionnaires [43]. Only if the participants selected the correct option from this choice would their questionnaire data be included in the subsequent analysis. The scheme of the orthogonal experiment is shown in Table 3. At the same time, according to the rule of thumb proposed by Johnson and Orme [44], the minimum sample size for a DCE study can be calculated by the formula:

**Table 3:** Results of the orthogonal experiment on the choice preference of the medical-elderly care integrated institutions

No.	Monthly Cost	Environmental Facilities	Service Quality	Medical Technology Level	Entertainment Activities	Convenience of Transportation
1	1	1	1	1	1	1
2	1	1	2	2	3	2
3	1	2	1	3	3	3
4	1	2	3	1	2	4
5	1	3	2	3	2	1
6	1	3	3	2	1	2
7	2	1	1	3	2	1
8	2	1	3	1	3	2
9	2	2	2	2	2	1
10	2	2	3	3	1	2
11	2	3	1	2	3	4
12	2	3	2	1	1	3
13	3	1	2	3	1	4
14	3	1	3	2	2	3
15	3	2	1	2	1	1
16	3	2	2	1	3	2
17	3	3	1	1	2	2
18	3	1	3	3	3	1

$$ \text{N}>500\frac{c}{(t\times a)}$$

where *N* represents the recommended minimum sample size, *t* signifies the number of selected tasks, *a* denotes the number of selections per task, and *c* represents the maximum number of attribute levels. In this study, *c* was 5, *a* was 2, and *t* was 16, so the minimum sample size was 78. However, 420 valid samples were included in this study, and the sample size was much larger than the minimum sample size, thus ensuring the accuracy and reliability of the experiment.

### Data collection

Due to the impact of the COVID-19 pandemic, the research team could not conduct a large-scale questionnaire survey, and the respondents were middle-aged and older people, so a web-based survey was not applicable. Therefore, from October 20, 2022, to November 10, 2022, the research team randomly selected three cities in the eastern, central, and western regions of China for the survey by multi-stage sampling and then selected three administrative districts/counties in each city by the random number table method, and finally identified Siming, Tongan and Huli districts in Xiamen, Fujian Province, Xuanzhou, Guangde and Jixi counties in Xuancheng City, Anhui Province, and Changle, Beilin and Weiyang districts in Xi’an City, Shaanxi Province, as survey sites. Through the recruitment of surveyors, the research team, based on the principle of convenience, randomly selected one urban community and one rural village in each administrative district/county. Surveys were conducted by visiting households, with a uniform distribution of 50 questionnaires in each area, totaling 450 questionnaires. The effective response rate was 93.33%, with 420 valid questionnaires collected.

Strict inclusion and exclusion criteria were developed for this study. The inclusion criteria were: ① age greater than or equal to 45 years old (According to the World Health Organization (WHO) criteria for the age of the population, people over 45 are identified as middle-aged and older.); ② no cognitive impairment; ③ Intention to choose institutional care for aging currently or in the future; the exclusion criteria were: ① the presence of a large number of blank questions; ② wrong selection of the lie detector questions. The first part of the questionnaire is demographic characteristics, including gender, age, marital status, location, education level, and basic medical insurance. The second part of the questionnaire is the preference of medical-elderly care integrated institutions: first, a representative choice set is presented to the participants, and reasonable answers are given to help them understand the design of DCE; second, 8 choice sets are put in the formal survey, and finally, the choice set with obvious advantages (lie detector questions) is put in. The third part of the questionnaire is the Activity of Daily Living scale, which was developed by the Lawton and Brody study [45] and includes 2 dimensions of physical independence of daily living (PADL) and instrumental ability of daily living (IADL), with a total of 14 items. Each item was scored on a 4-point Likert scale from “1 = completely able to do it by myself” to “4 = completely unable to do it by myself”, with a total score of 14–56. A score of ≤ 22 indicates normal independence, while > 22 indicates different degrees of impairment in independence, and the higher the score, the worse the independence (Questionnaire A and Questionnaire B). Informed consent was obtained from all participants and approved by the Medical Ethics Committee of Binzhou Medical University (2022 − 280). The study followed the reporting guidelines of the American Association for Public Opinion Research (AAPOR). Data analysis was conducted in November 2022.

### Data analysis

Descriptive statistics show the demographic characteristics and Activity of Daily Living (ADL) of middle-aged and elderly participants. Random Utility Theory (RUT) provides the theoretical basis for analyzing DCE data [46]. RUT is a theoretical framework used to explain the decision-making process of individuals when making choices. The core idea of this theory is that when people make choices among different options, they take into account their individual preferences and utility (satisfaction level), and these choices are not entirely deterministic but involve a degree of randomness. DCE data are binary, where a “1” indicates an option is selected and a “0” indicates another option is selected. The DCE data were analyzed using a mixed logit regression (MXL) model in STATA15, where dummy codes were utilized for the levels of all attributes except the cost to address the heterogeneity of the mixed logit model and the independence of the uncorrelated assumptions. The nlcom program was also used to calculate the willingness to pay (WTP) for a given level of variation in each scenario, WTP, derived from a DCE, efficiently quantifies the economic sacrifices individuals are willing to make when selecting one diagnosis attribute level over another, often referred to as the reference attribute level. which is the monetary value of the middle-aged and older people’s preferences for different attributes of the medical-elderly care integrated institutions.

## Results

### Participants

The socio-demographic characteristics of the present study are shown in Table 4. A total of 420 samples were collected in this study, of which 198 (47.14%) were female, 237 (56.43%) were middle-aged, 183 (43.57%) were elderly, more than 90% of the respondents had a partner (90.71%), 296 (70.48%) lived in urban areas, the majority had more than 6 years of education (72.86%), the majority of respondents had basic health insurance (97.86%), and 382 people (90.95%) have a high level of daily activities.

**Table 4:** Characteristics of the study sample (N = 420)

Variable	Respondents, No. (%)
Sex	
Female	198 (47.14)
Male	222 (52.86)
Age, y	
45–60	237 (56.43)
≥ 60	183 (43.57)
Marital Status	
Have a partner	381 (90.71)
No partner	39 (9.29)
Location	
Urban	296 (70.48)
Rural	124 (29.52)
Education Level	
Low (≤ 6 y)	114 (27.14)
Medium (6 to 9 y)	191 (45.48)
High (> 9 y)	115 (27.38)
Basic medical insurance	
Yes	411 (97.86)
No	9 (2.14)
Activity of Daily Living	
Low	38 (9.05)
High	382 (90.95)

### Logit model effect results

With the mixed logit model, we found that Δ*R*^*2*^ was 0.366, *p* < 0.001, indicating that the model fit was good, and all covariates were significant except (*p* < 0.005). Based on the preference weight in the mixed logit model in Table 5, moderate service quality (vs. poor service quality: *β* = 1.707, *p* < 0.001, 95% *CI* 1.343 ~ 2.071) and high medical technology level (vs. low medical technology level: *β* = 1.535, *p* < 0.001, 95% *CI* 1.240 ~ 1.830) were the most important attributes to middle-aged and older people, followed by monthly cost, environmental facilities, the convenience of transportation, and entertainment activities. The middle-aged and older people’s preferences for comfortable environmental facilities, better service quality, high medical technology level, abundant entertainment activities, highly convenient transportation, and lower consumption level were significantly associated with their choices for medical-elderly care integrated institutions, as shown in Table 5.

**Table 5:** Analysis of middle-aged and older people’s preference for medical-elderly care integrated institutions

Variable	*β*	SE	Z	*P*	95% *CI*
cost	-7.556*10^− 4^	2.564*10^− 4^	-2.95	0.003	-1.258*10^− 3^ ~ -2.530*10^− 4^
Environmental Facilities					
Poor	1 [Reference]	NA	NA	NA	NA
Moderate	0.998	0.160	6.22	< 0.001	0.684 ~ 1.312
Good	1.015	0.164	6.19	< 0.001	0.694 ~ 1.336
Service Quality					
Poor	1 [Reference]	NA	NA	NA	NA
Moderate	1.707	0.186	9.20	< 0.001	1.343 ~ 2.071
Good	1.558	0.178	8.74	< 0.001	1.209 ~ 1.909
Medical Technology Level					
Low	1 [Reference]	NA	NA	NA	NA
Moderate	1.374	0.185	7.43	< 0.001	1.012 ~ 1.737
High	1.535	0.151	10.20	< 0.001	1.240 ~ 1.830
Entertainment Activities					
Not rich	1 [Reference]	NA	NA	NA	NA
General	0.556	0.160	3.47	0.001	0.242 ~ 0.870
Very rich	1.019	0.130	7.82	< 0.001	0.764 ~ 1.274
Convenience of Transportation					
< 10 min	1 [Reference]	NA	NA	NA	NA
10 ~ 30 min	0.592	0.162	3.64	< 0.001	0.274 ~ 0.910
> 30 min	0.687	0.175	3.92	< 0.001	0.344 ~ 1.031
const	0.120	0.346	0.35	0.729	-0.566 ~ 0.798

Tables 6, 7, 8, 9 and 10 present the preference analysis of middle-aged and elderly individuals in medical-elderly care integrated institutions based on different demographic characteristics. From Table 6, it is observed that moderate service quality (vs. poor service quality: *β* = 1.467, *p* < 0.001, 95% *CI* 0.958 ~ 1.976) and high doctor technical level (vs. low doctor technical level: *β* = 1.316, *p* < 0.001, 95% *CI* 0.911 ~ 1.721) were the most crucial attributes for male middle-aged and elderly individuals. Following closely were environmental facilities, convenience of transportation, entertainment activities, and monthly expenses. Male middle-aged and elderly individuals exhibited a significant association with their preferences for comfortable environmental facilities, high service quality, advanced medical technology, fewer entertainment activities, very convenient transportation, and lower consumption levels. On the other hand, moderate service quality (vs. poor service quality: *β* = 1.897, *p* < 0.001, 95% *CI* 1.366 ~ 2.427) and high doctor technical level (vs. low doctor technical level: *β* = 1.748, *p* < 0.001, 95% *CI* 1.310 ~ 2.186) were the predominant attributes for female middle-aged and elderly individuals. However, they tended to favor lower consumption levels, poorer environmental facilities, extremely rich entertainment activities, and very convenient transportation. Table 7 reveals that middle-aged individuals leaned towards excellent environmental facilities, moderate service quality, high medical technology, lower entertainment activities, and convenient transportation. Meanwhile, elderly individuals inclined towards moderate service quality, moderate medical level, and lower consumption expenditures. From Table 8, it can be inferred that middle-aged and elderly individuals without partners leaned towards high-level medical technology, with no significant preferences for other attributes. Conversely, those with partners tended to prefer moderate environmental facilities, moderate service quality, moderate doctor technical level, abundant entertainment activities, convenient transportation, and lower consumption expenditures. Table 9 indicates that urban middle-aged and elderly individuals were inclined towards poorer environmental facilities, moderate service quality, moderate doctor technical level, extremely rich entertainment activities, and convenient transportation. In contrast, rural middle-aged and elderly individuals favored moderate environmental facilities, moderate service quality, high doctor technical level, fewer entertainment activities, convenient transportation, and lower consumption expenditures. Finally, Table 10 illustrates that middle-aged and elderly individuals with lower daily activity capabilities leaned towards high service quality and high doctor technical level, while those with better daily activity capabilities favored good environmental facilities, moderate service quality, high doctor technical level, extremely rich entertainment activities, and very convenient transportation.

**Table 6:** Preference analysis of medical-elderly care integrated institutions among middle-aged and elderly people with different gender characteristics

Variable	Female			Male		
	*β*	*P*	95%*CI*	*β*	*P*	95%*CI*
cost	-0.720*10^− 4^	0.044	-1.421*10^− 4^ ~ -1.871*10^− 5^	-0.794*10^− 4^	0.034	-1.525*10^− 4^~-6.176*10^− 5^
Environmental Facilities						
Poor	1 [Reference]	NA	NA	1 [Reference]	NA	NA
Moderate	1.047	< 0.001	0.603 ~ 1.492	0.906	< 0.001	0.453 ~ 1.360
Good	1.051	< 0.001	0.603 ~ 1.498	0.942	< 0.001	0.471 ~ 1.413
Service Quality						
Poor	1 [Reference]	NA	NA	1 [Reference]	NA	NA
Moderate	1.467	< 0.001	0.958 ~ 1.976	1.897	< 0.001	1.366 ~ 2.427
Good	1.414	< 0.001	0.939 ~ 1.890	1.643	< 0.001	1.117 ~ 2.170
Medical Technology Level						
Low	1 [Reference]	NA	NA	1 [Reference]	NA	NA
Moderate	0.985	< 0.001	0.475 ~ 1.494	1.735	< 0.001	1.207 ~ 2.263
High	1.316	< 0.001	0.911 ~ 1.721	1.748	< 0.001	1.310 ~ 2.186
Entertainment Activities						
Not rich	1 [Reference]	NA	NA	1 [Reference]	NA	NA
General	0.516	0.018	0.088 ~ 0.943	0.566	0.018	0.096 ~ 1.036
Very rich	0.943	< 0.001	0.583 ~ 1.303	1.077	< 0.001	0.710 ~ 1.445
Convenience of Transportation						
< 10 min	1 [Reference]	NA	NA	1 [Reference]	NA	NA
10 ~ 30 min	0.469	0.044	0.012 ~ 0.927	0.653	0.004	0.204 ~ 1.103
> 30 min	0.727	0.003	0.252 ~ 1.201	0.590	0.024	0.079 ~ 1.101
const	-0.177	0.718	-1.141 ~ 0.786	0.311	0.529	-0.658 ~ 1.281

**Table 7:** Preference analysis of medical-elderly care integrated institutions with different age characteristics

Variable	Age (45–59 y)			Age (≥ 60 y)		
	*β*	*P*	95%*CI*	*β*	*P*	95%*CI*
cost	-5.446*10^− 4^	0.859	-0.544*10^− 4^ ~ 0.653*10^− 4^	-3.069*10^− 4^	< 0.001	-4.220*10^− 4^~-1.918*10^− 4^
Environmental Facilities						
Poor	1 [Reference]	NA	NA	1 [Reference]	NA	NA
Moderate	0.918	< 0.001	0.533 ~ 1.303	0.548	0.123	-0.148 ~ 1.245
Good	1.053	< 0.001	0.657 ~ 1.449	0.276	0.432	-0.413 ~ 0.966
Service Quality						
Poor	1 [Reference]	NA	NA	1 [Reference]	NA	NA
Moderate	1.408	< 0.001	0.955 ~ 1.861	1.894	< 0.001	1.164 ~ 2.624
Good	1.382	< 0.001	0.950 ~ 1.813	1.536	< 0.001	0.808 ~ 2.263
Medical Technology Level						
Low	1 [Reference]	NA	NA	1 [Reference]	NA	NA
Moderate	1.035	< 0.001	0.576 ~ 1.493	1.488	< 0.001	0.821 ~ 2.155
High	1.411	< 0.001	1.043 ~ 1.776	1.482	< 0.001	0.940 ~ 2.025
Entertainment Activities						
Not rich	1 [Reference]	NA	NA	1 [Reference]	NA	NA
General	0.664	< 0.001	0.292 ~ 1.037	0.383	0.334	-1.158 ~ 0.393
Very rich	0.984	< 0.001	0.660 ~ 1.308	0.710	0.003	0.237 ~ 1.184
Convenience of Transportation						
< 10 min	1 [Reference]	NA	NA	1 [Reference]	NA	NA
10 ~ 30 min	0.594	0.003	0.207 ~ 0.981	0.065	0.860	-0.789 ~ 0.660
> 30 min	0.635	0.003	0.219 ~ 1.051	0.357	0.364	-0.414 ~ 1.129
const	-0.208	0.625	-1.042 ~ 0.626	-0.435	0.558	-1.892 ~ 1.021

**Table 8:** Preference analysis of medical-elderly care integrated institutions with different marital characteristics

Variable	No partner			Have a partner		
	*β*	*P*	95%*CI*	*β*	*P*	95%*CI*
cost	-1.487*10^− 4^	0.186	-3.693*10^− 4~^0.718*10^− 4^	-0.738*10 − 4	0.006	-1.259*10 − 4~-0.216*10 − 4
Environmental Facilities						
Poor	1 [Reference]	NA	NA	1 [Reference]	NA	NA
Moderate	0.225	0.757	-1.199 ~ 1.649	1.034	< 0.001	0.706 ~ 1.361
Good	0.796	0.273	-0.627 ~ 2.219	1.012	< 0.001	0.680 ~ 1.345
Service Quality						
Poor	1 [Reference]	NA	NA	1 [Reference]	NA	NA
Moderate	1.033	0.165	-0.424 ~ 2.489	1.742	< 0.001	1.363 ~ 2.121
Good	1.454	0.077	-0.159 ~ 3.068	1.541	< 0.001	1.181 ~ 1.901
Medical Technology Level						
Low	1 [Reference]	NA	NA	1 [Reference]	NA	NA
Moderate	0.799	0.274	-0.632 ~ 2.230	1.406	< 0.001	1.029 ~ 1.783
High	1.676	0.004	0.528 ~ 2.825	1.521	< 0.001	1.215 ~ 1.828
Entertainment Activities						
Not rich	1 [Reference]	NA	NA	1 [Reference]	NA	NA
General	-0.432	0.604	-2.067 ~ 1.203	0.591	< 0.001	0.268 ~ 0.914
Very rich	1.024	0.049	0.094 ~ 2.049	1.007	< 0.001	0.741 ~ 1.273
Convenience of Transportation						
< 10 min	1 [Reference]	NA	NA	1 [Reference]	NA	NA
10 ~ 30 min	-0.389	0.556	-1.684 ~ 0.907	0.657	< 0.001	0.324 ~ 0.991
> 30 min	-0.680	0.410	-2.297 ~ 0.937	0.763	< 0.001	0.405 ~ 1.120
const	-2.460	0.101	-5.399 ~ 0.478	0.280	0.436	-0.426 ~ 0.987

**Table 9:** Preference analysis of medical-elderly care integrated institutions with different location characteristics

Variable	Urban			Rural		
	*β*	*P*	95%*CI*	*β*	*P*	95%*CI*
cost	0.397*10^− 5^	0.993	-0.863*10^− 4^~0.871*10^− 4^	-1.141*10^− 4^	< 0.001	-1.768*10^− 4^~-0.515*10^− 4^
Environmental Facilities						
Poor	1 [Reference]	NA	NA	1 [Reference]	NA	NA
Moderate	0.731	0.012	0.159 ~ 1.304	1.146	< 0.001	0.761 ~ 1.531
Good	0.942	0.002	0.354 ~ 1.530	1.077	< 0.001	0.688 ~ 1.465
Service Quality						
Poor	1 [Reference]	NA	NA	1 [Reference]	NA	NA
Moderate	1.715	< 0.001	1.046 ~ 2.385	1.719	< 0.001	1.279 ~ 2.158
Good	1.479	< 0.001	0.846 ~ 2.112	1.604	< 0.001	1.179 ~ 2.028
Medical Technology Level						
Low	1 [Reference]	NA	NA	1 [Reference]	NA	NA
Moderate	1.297	< 0.001	0.631 ~ 1.962	1.443	< 0.001	1.006 ~ 1.880
High	1.237	< 0.001	0.701 ~ 1.774	1.694	< 0.001	1.335 ~ 2.052
Entertainment Activities						
Not rich	1 [Reference]	NA	NA	1 [Reference]	NA	NA
General	0.517	0.073	-0.047 ~ 1.082	0.579	0.003	0.198 ~ 0.959
Very rich	1.141	< 0.001	0.669 ~ 1.612	0.949	< 0.001	0.643 ~ 1.256
Convenience of Transportation						
< 10 min	1 [Reference]	NA	NA	1 [Reference]	NA	NA
10 ~ 30 min	0.701	0.021	0.105 ~ 1.298	0.580	0.003	0.198 ~ 0.962
> 30 min	0.995	0.002	0.369 ~ 1.620	0.567	0.008	0.148 ~ 0.986
const	-0.212	0.734	-1.433 ~ 1.009	0.325	0.441	-0.503 ~ 1.153

**Table 10:** Preference analysis of medical-elderly care integrated institutions with different activity of daily living characteristics

Variable	Activity of Daily Living (Low)			Activity of Daily Living (High)		
	*β*	*P*	95%*CI*	*β*	*P*	95%*CI*
cost	-1.565*10^− 4^	0.158	-3.738*10^− 4^~0.690*10^− 4^	-0.688*10^− 4^	0.009	-1.207*10^− 4^~-0.168*10^− 4^
Environmental Facilities						
Poor	1 [Reference]	NA	NA	1 [Reference]	NA	NA
Moderate	0.767	0.194	-0.391 ~ 1.925	1.041	< 0.001	0.710 ~ 1.371
Good	0.444	0.442	-0.689 ~ 1.577	1.073	< 0.001	0.736 ~ 1.410
Service Quality						
Poor	1 [Reference]	NA	NA	1 [Reference]	NA	NA
Moderate	1.608	0.022	0.232 ~ 2.984	1.723	< 0.001	1.343 ~ 2.102
Good	1.812	0.006	0.526 ~ 3.099	1.527	< 0.001	1.162 ~ 1.892
Medical Technology Level						
Low	1 [Reference]	NA	NA	1 [Reference]	NA	NA
Moderate	1.800	0.006	0.520 ~ 3.080	1.337	< 0.001	0.957 ~ 1.718
High	1.932	0.001	0.816 ~ 3.047	1.503	< 0.001	1.196 ~ 1.810
Entertainment Activities						
Not rich	1 [Reference]	NA	NA	1 [Reference]	NA	NA
General	0.901	0.132	-0.272 ~ 2.073	0.523	0.002	0.196 ~ 0.850
Very rich	0.950	0.060	-0.038 ~ 1.939	1.017	< 0.001	0.751 ~ 1.283
Convenience of Transportation						
< 10 min	1 [Reference]	NA	NA	1 [Reference]	NA	NA
10 ~ 30 min	1.102	0.055	-0.025 ~ 2.228	0.557	0.001	0.220 ~ 0.893
> 30 min	0.335	0.621	-0.991 ~ 1.661	0.721	< 0.001	0.362 ~ 1.081
const	0.867	0.512	-1.726 ~ 3.460	0.072	0.843	-0.639 ~ 0.782

### Willingness to pay (WTP)

Table 11 shows the different levels of WTP for specific attributes, and the results of WTP are a monetary measure of participants’ preference for medical-elderly care integrated institutions. It was found that participants were more willing to pay for the service quality and the medical technology level than for the other attributes. They are willing to pay an additional $3156 and $2838 to improve “poor service quality” and “poor doctor skill level” to " moderate service quality " (*p* = 0.007, 95% *CI* 963 ~ 5349) and “high medical technology” (*p* = 0.005, 95% *CI* 852 ~ 4824). In terms of the convenience of transportation, participants were willing to pay only $1094 and $1271 to change the time to walk from the nursing home to the nearest bus stop from “>30min” to “10-30min” (*p* = 0.020, 95% *CI* 173 ~ 2015) and “<10 min” (*p* = 0.016, 95% CI 235 ~ 2308). According to the WTP results in Table 11, the maximum WTP for the best medical-elderly care integrated institutions choice option (moderate service quality, high medical technology level, good environmental facilities, very rich entertainment activities, and < 10 min to walk to the nearest bus stop) was $11,025.

**Table 11:** Willingness to pay for level changes of specific attributes

Variable	Willingness to pay, $^*a*^	SE	Z	*P*	95% *CI*
Environmental Facilities					
Poor	1 [Reference]	NA	NA	NA	NA
Moderate	1845	673	2.74	0.006	525 ~ 3165
Good	1876	744	2.52	0.012	419 ~ 3334
Service Quality					
Poor	1 [Reference]	NA	NA	NA	NA
Moderate	3156	1119	2.82	0.005	963 ~ 5349
Good	2881	1061	2.71	0.007	801 ~ 4961
Medical Technology Level					
Low	1 [Reference]	NA	NA	NA	NA
Moderate	2540	926	2.74	0.006	726 ~ 4354
High	2838	1013	2.80	0.005	852 ~ 4824
Entertainment Activities					
Not rich	1 [Reference]	NA	NA	NA	NA
General	1028	462	2.22	0.026	122 ~ 1934
Very rich	1884	761	2.48	0.013	3925 ~ 3375
Convenience of Transportation					
> 30 min	1 [Reference]	NA	NA	NA	NA
10 ~ 30 min	1094	470	2.33	0.020	173 ~ 2015
< 10 min	1271	529	2.40	0.016	235 ~ 2308

^*a*^ Based on a currency exchange rate of 7.1598 yuan to $1.00 in November 2022

## Discussion

This study reports the results of a DCE study quantifying the preferences of middle-aged and older people for medical-elderly care integrated institutions. The study shows that middle-aged and older people preferred an institution with moderate service quality, a high medical technology level, good environmental facilities, very rich entertainment activities, and a < 10 min walk to the nearest bus stop. Moreover, they had a greater WTP for improving service quality from a poor to a moderate level than for extending entertainment activities or improving the convenience of transportation.

Our study found that better service quality was the highest priority for middle-aged and older people. Middle-aged and older people are willing to pay the highest amount ($3156) to get improved service quality, and when the service quality increases from poor to a moderate level, the WTP increases by $3156, which is higher than the WTP from poor to a good level ($ 2881). The average payment for medical-elderly care integrated institutions in China ranges from $6,857 to $51,428 annually [47]. Older adults in China are willing to increase their payments by $3,156 to enhance the service quality of medical-elderly care integrated institutions, indicating their significantly high expectations for service quality. Currently, there is a lack of uniform standards and regulations for service standards and quality management in Chinese medical-elderly care integrated institutions, leading to unstable service quality and making it difficult to ensure that residents receive high-level medical and elderly care services [48]. Due to the dual nature of medical and elderly care services provided by these institutions, there is a disparity in service levels. Some institutions may excel in medical services while performing relatively weakly in elderly care services. Moreover, significant variations exist in hardware facilities and staff qualifications among different regions and types of medical-elderly care integrated institutions. These disparities prompt middle-aged and elderly individuals to seek better service quality [49]. Studies related to humanistic theory and elderly service needs concluded that there are layers, multiplicity, and hierarchy in elderly service needs [50]. Regarding basic physiological and material needs, some studies have found a relatively high proportion of disabled and semi-disabled older people in elderly care institutions and that chronic diseases and health monitoring need continuous attention as their physical functions deteriorate [51]. Therefore, integrated medical-elderly care institutions need not only to provide good daily care services but also to strengthen the health management of older people to prevent the occurrence of diseases. At the same time, related studies have also found that older people have a high prevalence of psychological diseases and are in greater need of spiritual comfort, while elderly care institutions often lack attention to spiritual services [52]. Therefore, there is a need to individually set up spiritual support according to the elderly’s characteristics to expand their relationships and improve emotional support. However, there is an overall low quality of nursing staff, a lack of personnel, and an unbalanced ratio of older people and nursing staff in Chinese elderly care institutions [22], which leads to an urgent need for middle-aged and older people to improve the service quality of elderly care institutions, but a relatively low pursuit of a very high level of service quality.

Our study revealed that middle-aged and elderly individuals exhibit a stronger preference for high-level medical technology. Elderly individuals, being a high-risk group for chronic diseases, possess physical vulnerability and psychological sensitivity [53]. Their disease management involves complexity, suddenness, and persistence [54–55], necessitating a substantial tilt towards high-level medical resources. As carriers of both medical and elderly care services, medical-elderly care integrated institutions primarily combine the medical expertise, rehabilitation training, and elderly care provided by hospitals. This integration effectively ensures the quality of life for older adults in their later years. The core of the development of these institutions lies in the level of medical technology [14], requiring individuals with dual professional knowledge in medicine and nursing. However, the current state of medical facilities, severe shortages in medical and nursing staff, and an imperfect talent cultivation system in Chinese medical-elderly care integrated institutions pose significant challenges. Due to substantial differences in the services and work environments provided by hospitals and medical-elderly care integrated institutions, and the more mature professional development system in hospitals, medical and nursing staff tend to prefer working in hospital environments dominated by treatment and medicine. This shortage is particularly pronounced among highly skilled professionals, such as doctors. Consequently, middle-aged and elderly individuals are inclined towards institutions with higher-level doctors and nursing staff to ensure their health and safety in medical-elderly care integrated institutions. Therefore, these institutions should prioritize the cultivation of medical-elderly care service professionals. This can be achieved through regular systematic and professional training in medical-elderly care knowledge, inviting medical and nursing experts for technical guidance to enhance the service quality of medical-elderly care integrated institutions. Simultaneously, it is crucial to improve the management and assessment system for medical and nursing staff, clarify their professional development directions, and enhance their motivation to establish a high-level medical technology team.

Our results also show that middle-aged and older people prefer medical-elderly care integrated institutions to have good environmental facilities. In general, older people and their families consider the living experience when choosing an institution, including the comfort, safety, and convenience of living, whether the physical facilities can meet individual needs, and whether they have privacy space [56]. However, at present, most of the medical-elderly care integrated institutions in China are more from the perspective of self-development and profitability. To some extent, they ignore the needs of their users, and there are problems such as small space size, a large number of people served, and inadequate medical equipment [33]. Therefore, it is necessary to understand and consider the actual needs of the elderly fully, adhering to the construction concept of solving problems for the elderly, improving their quality of life and living standards, and establishing a more comfortable and warm living environment for the elderly. At the same time, the spiritual and cultural needs of the elderly should be fully considered. Space facilities, including chess and card rooms, fitness and rehabilitation rooms, and entertainment rooms, should be established to regularly carry out entertainment activities and involve the elderly as much as possible to improve their subjective sense of well-being and quality of life.

We found that middle-aged and elderly individuals tend to prefer lower payment fees, aligning with previous research results [47]. This inclination may be attributed to significant variations in pricing among medical-elderly care integrated institutions at present, and related studies have identified pricing in high-quality medical-elderly care integrated institutions to be several times higher than that of conventional elderly care institutions [57]. The majority of the elderly population in China exhibits lower economic independence and risk resilience. Due to economic constraints, they are unable to bear the substantial financial burden associated with institutional elderly care [47]. Secondly, as the older adults’ muscles continue to weaken, their demand for health care services increases, further increasing the cost of long-term care. Even in most developed countries, many older people cannot afford continuous, high-level care if they rely solely on government support and individual payments [58]. Therefore, actively exploring and developing long-term care insurance is particularly important. It not only reduces the burden on the elderly and their families but also serves as an essential source of funding for the long-term development of medical-elderly care integrated institutions. Medical insurance is the bearer of disease risk for the elderly, long-term care insurance is the guardian of continuous care for the elderly, and commercial insurance supplements long-term care insurance. The three complement each other to provide security for a happy and safe old age. Furthermore, it is interesting to note that there are variations in the preferences of middle-aged and elderly individuals with different demographic characteristics for medical-elderly care integrated institutions. For instance, elderly individuals with lower daily activity capacity tend to lean towards higher service quality and advanced medical technology. In contrast, those with better daily activity capacity prefer good environmental facilities, moderate service quality, advanced medical technology, a wide range of recreational activities, and extremely convenient transportation. Due to physical limitations, elderly individuals with lower daily activity capacity seek greater attention to their physical well-being, while those with better daily activity capacity aspire to have enriched life care. This suggests the importance of providing personalized care services tailored to the diverse needs of different middle-aged and elderly individuals.

Therefore, for top-level decision-makers, government authorities can enact relevant policies and regulations to specify the requirements for medical-elderly care integrated institutions, including service standards, staffing, and facility provisions. This can contribute to elevating industry standardization and safeguarding the legitimate rights of the elderly. Additionally, establishing a comprehensive talent development system for medical-elderly care integration is essential, encouraging cross-training between medical and elderly care personnel to enhance their professional competence. Government entities can implement incentive policies to attract more healthcare professionals and caregivers to join medical-elderly care integrated institutions. Simultaneously, financial subsidies and tax incentives can be provided to support the construction and operation of these institutions, thereby alleviating their economic burden and improving service levels. Furthermore, encouraging and supporting the participation of social forces and enterprises in the construction of medical-elderly care integrated institutions is crucial. The government can offer policy support, including land policies and financing support, to promote the development of more high-quality medical-elderly care integrated institutions. Regarding the institutions themselves, there is a need to enhance service quality and medical standards. Firstly, ensuring that medical-elderly care integrated institutions are equipped with a professional nursing team, including registered nurses and nursing aides, is paramount. This team should possess specialized knowledge and nursing skills tailored to address various health issues faced by the elderly. Secondly, personalized care plans should be developed based on the health conditions and needs of each middle-aged and elderly individual. This involves regular health assessments, the formulation of nutrition and rehabilitation plans, ensuring comprehensive and thoughtful care. Establishing close cooperation with medical institutions to integrate healthcare resources is essential. Providing regular health check-ups, chronic disease management, medical rehabilitation, and other services for middle-aged and elderly individuals ensures effective management of their physical health. For those with lower daily living capabilities, rehabilitation nursing services, including physical therapy and occupational therapy, should be offered to assist in their recovery and enhance their quality of life. Regular assessments of their rehabilitation progress and adjustments to the rehabilitation plan to accommodate changes in their health status are crucial. Additionally, conducting health education activities to increase awareness of health among middle-aged and elderly individuals, encouraging active participation in disease prevention, and promoting healthy lifestyles can contribute to reducing the occurrence and progression of chronic diseases. Finally, establishing a nursing quality management mechanism with regular quality assessments and improvements, along with continuous training and learning for the nursing team to ensure the mastery of the latest nursing knowledge and skills, is imperative to enhance service levels.

## Limitations

This study has limitations. First, subject to attribute and number-of-levels limitations, the DCE may not represent all complex real-life preferences of middle-aged and older people for choosing medical-elderly care integrated institutions. Although this study selected three cities in east, central, and west China and included 420 middle-aged and older people, only three cities cannot fully measure the whole of China, and future studies can further expand the sample size.

## Conclusions

This study has provided essential insights into the characteristics of medical-elderly care integrated institutions that middle-aged and older people most value. The results suggest that more attention should be paid to the service quality and medical technology of medical-elderly care integrated institutions so that all middle-aged and older people can enjoy a high quality of life later. The state should attach great importance to the development of the medical-elderly care integrated service industry, actively optimize the medical-elderly care integrated service model, improve the physical facilities of the elderly care institutions, and create an excellent ecological environment, and at the same time, give full play to the role of medical insurance, long-term care insurance, and commercial insurance, to improve the overall quality of life of the elderly.

### Electronic supplementary material

Below is the link to the electronic supplementary material.

Supplementary Material 1

Supplementary Material 2

Supplementary Material 3

## Data Availability

The data sets used and/or analyzed during the current study are available from the corresponding author on reasonable request.
