# Supplementary material for: Middle-aged and older people’s preference for medical-elderly care integrated institutions in China: a discrete choice experiment study
Source: BMC Nurs. 2024 Jan 10;23:32. doi: 10.1186/s12912-023-01696-w (PMC10777634; doi:10.1186/s12912-023-01696-w)
Supplement: Supplementary file 2 — Supplementary Material 2 [file 12912_2023_1696_MOESM2_ESM.docx]

**Survey on Willingness and Preference for Medical-elderly Care Integrated Institutions (Volume B)**

Dear participants

Hello! We are conducting a study on the wishes and preferences of middle-aged and elderly people for medical-elderly care integrated institutions. This questionnaire is designed to understand some of your basic information, daily living abilities and preferences for Medical-elderly care integrated institutions. Your participation will help us better understand the needs of middle-aged and elderly people for integrated medical and elderly care institutions, and provide a reference for improving the service quality of integrated medical and elderly care institutions and enriching the content of integrated medical and elderly care institutions. Please take approximately 20 minutes to fill out this questionnaire. All data will be kept strictly confidential and will be used for academic research only.

Thank you for your support and participation!

1. If you agree to participate in our survey, please click Agree below and start the survey.

□Agree □Disagree (please skip to the end of the questionnaire and submit your answer sheet)

**(1) Basic information**

2. Gender: □ Male □ Female

3. Place of Birth: Province

4. Age:

5. Marital status: □ Married □ Widowed □ Divorced □ Single

6.Residence: □Rural □Urban

7. Years of education: □ ≤6 year □ 6 to 9 year □>9 year

8. Your medical insurance is: □ Urban employee medical insurance □ Urban and rural residents medical insurance □ None □ Others

**(2)** **Willingness and preference of medical-elderly care integrated institutions**

9. Which of the following two medical-elderly care integrated institutions models will you choose?

| Attributes | Choice Set A | Choice Set B |
| --- | --- | --- |
| Environmental Facilities | Moderate | Moderate |
| Service Quality | Moderate | Good |
| Medical technology level | Moderate | Poor |
| Entertainment Activities | General | General |
| Convenience of transportation | 10~30min | <10min |
| Monthly Cost | $297 | $1117 |
| Which one is more attractive | □ | □ |

10. Which of the following two medical-elderly care integrated institutions models will you choose?

| Attributes | Choice Set A | Choice Set B |
| --- | --- | --- |
| Environmental Facilities | Moderate | Moderate |
| Service Quality | Moderate | Good |
| Medical technology level | Moderate | Moderate |
| Entertainment Activities | General | Nor rich |
| Convenience of transportation | 10~30min | >30min |
| Monthly Cost | $297 | $838 |
| Which one is more attractive | □ | □ |

11. Which of the following two medical-elderly care integrated institutions models will you choose?

| Attributes | Choice Set A | Choice Set B |
| --- | --- | --- |
| Environmental Facilities | Moderate | Good |
| Service Quality | Moderate | Poor |
| Medical technology level | Moderate | Moderate |
| Entertainment Activities | General | Very rich |
| Convenience of transportation | 10~30min | >30min |
| Monthly Cost | $297 | $1117 |
| Which one is more attractive | □ | □ |

12. Which of the following two medical-elderly care integrated institutions models will you choose?

| Attributes | Choice Set A | Choice Set B |
| --- | --- | --- |
| Environmental Facilities | Moderate | Good |
| Service Quality | Moderate | Poor |
| Medical technology level | Moderate | Good |
| Entertainment Activities | General | General |
| Convenience of transportation | 10~30min | 10~30min |
| Monthly Cost | $297 | $838 |
| Which one is more attractive | □ | □ |

13. Which of the following two medical-elderly care integrated institutions models will you choose?

| Attributes | Choice Set A | Choice Set B |
| --- | --- | --- |
| Environmental Facilities | Moderate | Good |
| Service Quality | Moderate | Moderate |
| Medical technology level | Moderate | Poor |
| Entertainment Activities | General | General |
| Convenience of transportation | 10~30min | >30min |
| Monthly Cost | $297 | $297 |
| Which one is more attractive | □ | □ |

14. Which of the following two medical-elderly care integrated institutions models will you choose?

| Attributes | Choice Set A | Choice Set B |
| --- | --- | --- |
| Environmental Facilities | Moderate | Good |
| Service Quality | Moderate | Moderate |
| Medical technology level | Moderate | Moderate |
| Entertainment Activities | General | Nor rich |
| Convenience of transportation | 10~30min | <10min |
| Monthly Cost | $297 | $559 |
| Which one is more attractive | □ | □ |

15. Which of the following two medical-elderly care integrated institutions models will you choose?

| Attributes | Choice Set A | Choice Set B |
| --- | --- | --- |
| Environmental Facilities | Moderate | Good |
| Service Quality | Moderate | Good |
| Medical technology level | Moderate | Poor |
| Entertainment Activities | Moderate | Nor rich |
| Convenience of transportation | 10~30min | 10~30min |
| Monthly Cost | $297 | $559 |
| Which one is more attractive | □ | □ |

16. Which of the following two medical-elderly care integrated institutions models will you choose?

| Attributes | Choice Set A | Choice Set B |
| --- | --- | --- |
| Environmental Facilities | Moderate | Good |
| Service Quality | Moderate | Poor |
| Medical technology level | Moderate | Good |
| Entertainment Activities | Moderate | Very rich |
| Convenience of transportation | 10~30min | <10min |
| Monthly Cost | $297 | $297 |
| Which one is more attractive | □ | □ |

17. Which of the following two medical-elderly care integrated institutions models will you choose?

| Attributes | Choice Set A | Choice Set B |
| --- | --- | --- |
| Environmental Facilities | Moderate | Poor |
| Service Quality | Moderate | Poor |
| Medical technology level | Moderate | Poor |
| Entertainment Activities | Moderate | Nor rich |
| Convenience of transportation | 10~30min | >30min |
| Monthly Cost | $297 | $1117 |
| Which one is more attractive | □ | □ |

**(3) Activity of Daily Living**

18. Choose the situation that best suits you from the following

| Use public vehicles | □Can do it by myself □Some difficulty □Need help □Can’t do it at all |
| --- | --- |
| walk | □Can do it by myself □Some difficulty □Need help □Can’t do it at all |
| cook meals | □Can do it by myself □Some difficulty □Need help □Can’t do it at all |
| Do housework | □Can do it by myself □Some difficulty □Need help □Can’t do it at all |
| take medicine | □Can do it by myself □Some difficulty □Need help □Can’t do it at all |
| Cook | □Can do it by myself □Some difficulty □Need help □Can’t do it at all |
| dressing | □Can do it by myself □Some difficulty □Need help □Can’t do it at all |
| Comb your hair and brush your teeth | □Can do it by myself □Some difficulty □Need help □Can’t do it at all |
| Laundry | □Can do it by myself □Some difficulty □Need help □Can’t do it at all |
| bath | □Can do it by myself □Some difficulty □Need help □Can’t do it at all |
| Shopping | □Can do it by myself □Some difficulty □Need help □Can’t do it at all |
| Go to the toilet regularly | □Can do it by myself □Some difficulty □Need help □Can’t do it at all |
| Call up | □Can do it by myself □Some difficulty □Need help □Can’t do it at all |
| Handle your own money | □Can do it by myself □Some difficulty □Need help □Can’t do it at all |
