# Supplementary material for: Middle-aged and older people’s preference for medical-elderly care integrated institutions in China: a discrete choice experiment study
Source: BMC Nurs. 2024 Jan 10;23:32. doi: 10.1186/s12912-023-01696-w (PMC10777634; doi:10.1186/s12912-023-01696-w)
Supplement: Supplementary file 3 — Supplementary Material 3 [file 12912_2023_1696_MOESM3_ESM.docx]

**Expert interview outline on selection intention and preference of medical-elderly care integrated institutions**

Question1. Do you think middle-aged and elderly people will pay attention to environmental facilities when choosing medical-elderly Care Integrated Institutions? What aspects of environmental facilities will you pay attention to?

Question2. Do you think middle-aged and elderly people will pay attention to the service quality of nursing staff when choosing medical-elderly Care Integrated Institutions? What criteria are considered for service quality?

Question3. Do you think middle-aged and elderly people will pay attention to the technical level of doctors when choosing medical-elderly Care Integrated Institutions? How to properly measure the technical level of doctors?

Question4. Do you think middle-aged and elderly people will pay attention to rehabilitation activities or recreational activities when choosing medical-elderly Care Integrated Institutions? What levels of rehabilitation or recreational activities are appropriate?

Question5. Do you think middle-aged and elderly people will pay attention to transportation convenience when choosing medical-elderly Care Integrated Institutions? How is transportation convenience measured?

Question6. Do you think the average monthly cost for middle-aged and elderly people living in medical-elderly Care Integrated Institutions is very high, relatively high, low, or very low?

Question7. What aspects do you think middle-aged and elderly people should pay attention to when choosing medical-elderly Care Integrated Institutions?
